# Herpes Zoster Co-Infection in an Immunocompetent Patient With COVID-19

**DOI:** 10.7759/cureus.8998

**Published:** 2020-07-04

**Authors:** Ahmed Saati, Faisal Al-Husayni, Afnan A Malibari, Anas A Bogari, Maher Alharbi

**Affiliations:** 1 Internal Medicine, National Guard Hospital, Jeddah, SAU; 2 Internal Medicine, National Guard Hospital, King Abdulaziz Medical City, Jeddah, SAU; 3 Internal Medicine, King Saud Bin Abdulaziz University for Health Sciences, Jeddah, SAU; 4 Infection Prevention and Control, King Abdulaziz Medical City, Jeddah, SAU; 5 College of Medicine, King Saud Bin Abdulaziz University for Health Sciences, Jeddah, SAU; 6 Infectious Diseases, King Abdullah International Medical Research Center, Jeddah, SAU

**Keywords:** co-infection, corona virus disease, covid-19, famciclovir, herpes zoster, immunocompetent, immunocompetent adults, novel corona virus, sars-cov2, varicella-zoster virus

## Abstract

Severe acute respiratory syndrome coronavirus 2 (SARS-CoV-2) has a broad spectrum of manifestations. A variety of dermatological manifestations were described. We present a case of an immunocompetent middle-aged man who presented with novel coronavirus disease 2019 (COVID-19) and later developed herpes zoster (HZ). The case highlights the possibility of COVID-19-related HZ. The highest infection control measures must be abided when managing patients with cutaneous complaints until COVID-19 is ruled out.

## Introduction

Severe acute respiratory syndrome coronavirus 2 (SARS-CoV-2) was identified in December 2019 as the cause of COVID-19 in Wuhan City in Hubei Province, China. The high infectivity and the rapid transmission characteristics of the virus led to an epidemic throughout China initially, followed by a pandemic that is impacting a large number of people all over the world [1]. Initially, the transmission of SARS-CoV2 was considered a zoonotic transmission associated with the seafood market in Wuhan, China. Later on, human to human transmission through respiratory droplets and secretions was recognized to play a major role in the significant outbreak [1]. SARS-CoV2 is an enveloped, single-stranded RNA virus that belongs to the coronavirus family [2]. Cell entry is believed to be through the angiotensin-converting enzyme 2 (ACE2) receptors found on the surface of the cells [2]. Patients with COVID-19 can be asymptomatic or may show a range of mild, moderate, or severe symptoms, and may eventually lead to mortality. According to the Chinese Center for Disease Control and Prevention (CCDC), mild pneumonia appears to be the most common manifestation (81%). In comparison, around 14% had severe hypoxia and dyspnea, and about 5% critically developed acute respiratory distress syndrome (ARDS), shock, and multi-organ dysfunction [3]. A variety of dermatological manifestations were documented in COVID-19 cases. Morbilliform rashes or maculopapular exanthema were the most common, followed by a papulovesicular rash, urticaria, and other cutaneous signs [2]. Herpes zoster (HZ) is an acute, viral infection that occurs after the reactivation of the Varicella-zoster virus (VZV). The virus usually remains dormant within dorsal root ganglia after the virus's initial exposure in the form of varicella [4]. HZ probably appears when the immune system fails to contain the latent VZV replication. Therefore, it often occurs in the elderly, HIV-infected patients, and is more frequent in severely immunocompromised patients. Other factors, such as trauma, radiation, certain medications, and stress, can also trigger HZ but have not been determined with certainty [5]. In this report, we present a case of an immunocompetent adult who was admitted due to COVID-19 and later exhibited symptoms of HZ infection.

## Case presentation

**Figure 1:**
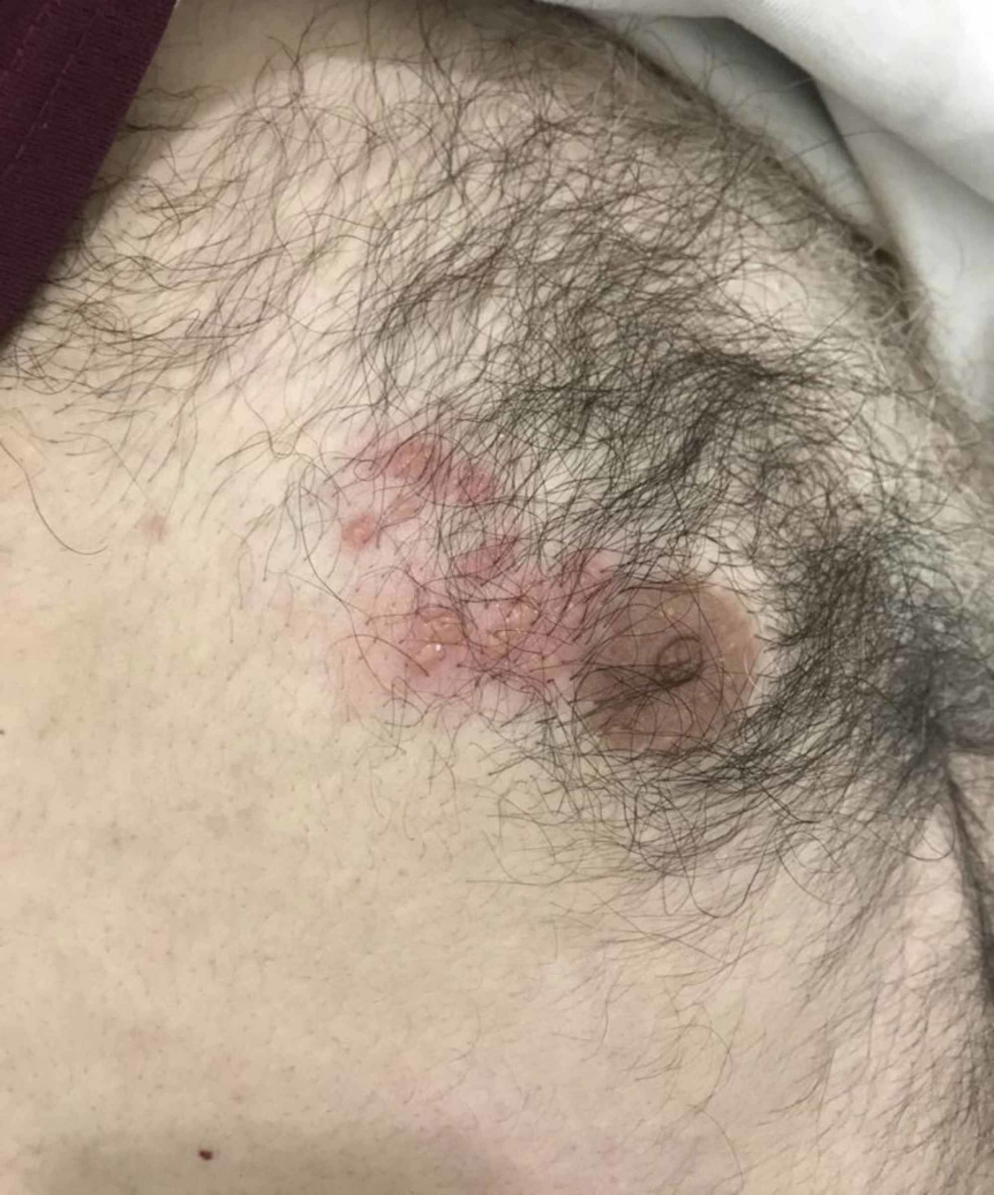
Vesicles with surrounding erythema affecting the area around the right nipple.

A 57-year-old male, known to have hypertension and VZV infection in childhood, presented with a four-day history of dry cough, shortness of breath, chills, headache, and loss of smell and taste. COVID-19 screening was initiated, and the patient was sent for home isolation. The next day, his nasopharyngeal swab turned positive for SARS-CoV2, and the patient was called back for admission. On presentation, the patient reported a sudden onset itch and painful rash that started after arriving home. The patient described the rash as fluid-filled bubbles that rupture upon scratching, releasing clear discharges. Vital signs and physical examination were normal except vesicles with surrounding erythema affecting the area around the right nipple (Figure 1).

**Figure 2:**
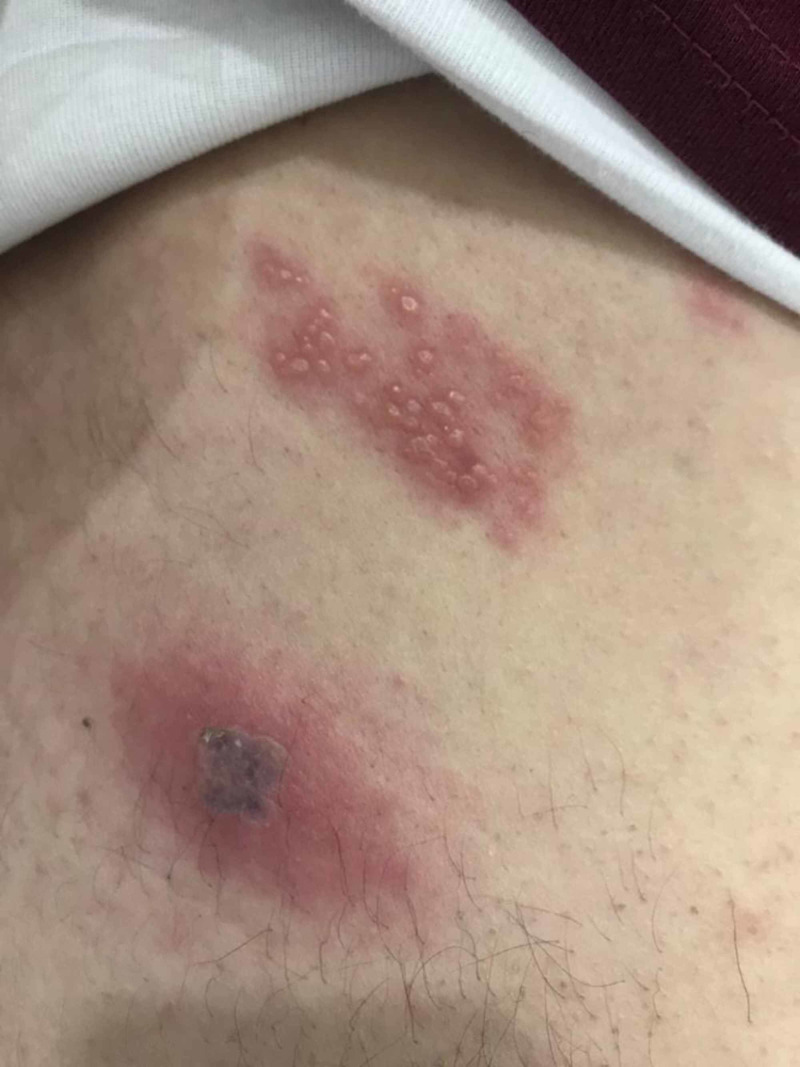
Vesicles on the tip of the right scapula with an area of scarring formed due to scratching, distribution is consistent with T4 dermatome.

Further, inspection revealed a similar rash on the tip of the right scapula with an area of scarring formed due to scratching the vesicles (Figure 2)..

**Figure 3:**
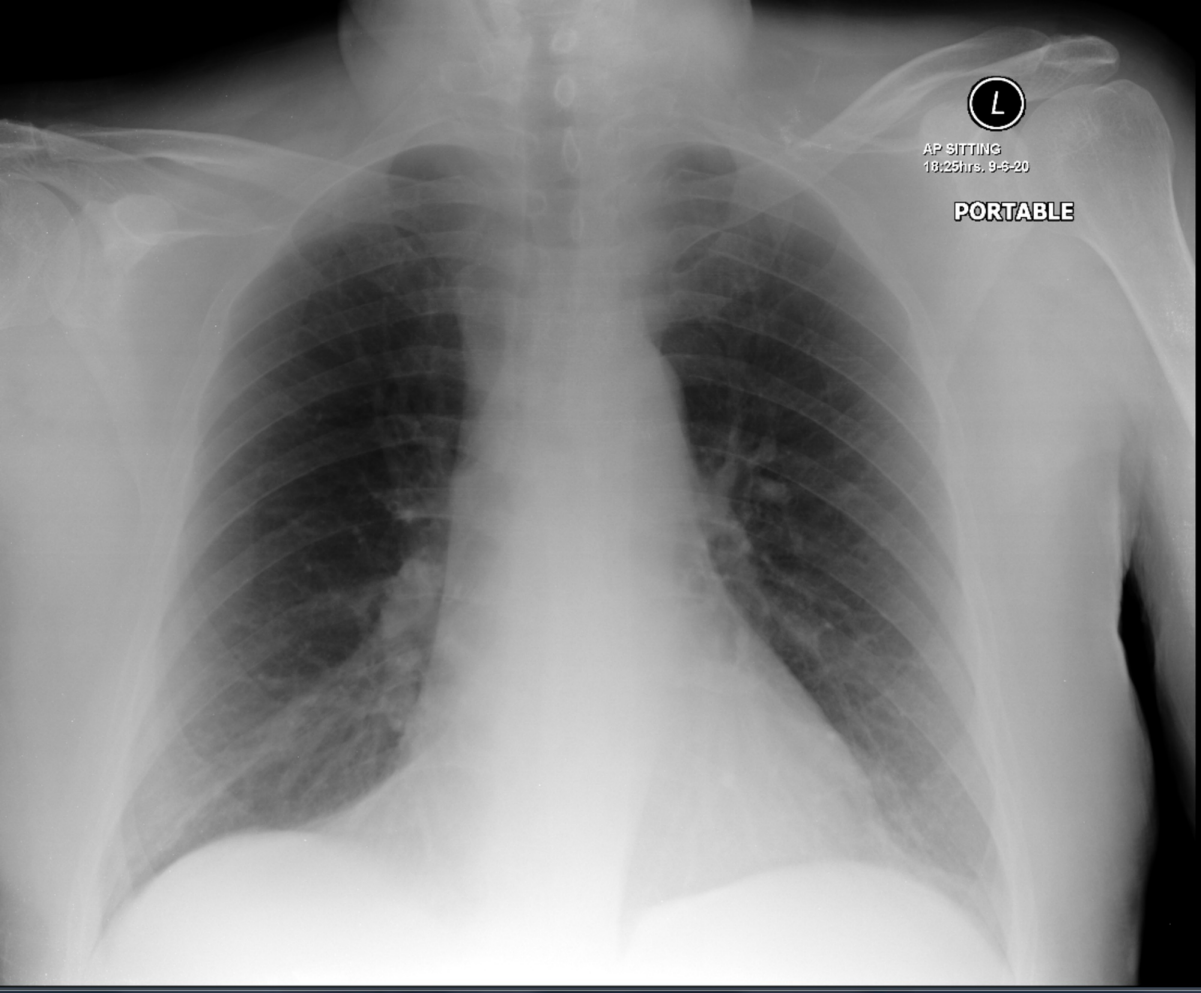
Patient’s chest X-ray showing normal appearance.

The spread of the rash is consistent with T4 dermatome, unilateral on the right side, with no extension over the midline supporting the diagnosis of HZ. Initial blood tests were unremarkable apart from slightly elevated C-reactive protein (5.2 mg/L) and estimated sedimentation rate (34 mm/h). A chest X-ray was obtained and had no significant findings (Figure 3).

HIV-serology test was negative, and immunoglobulin levels were normal. The patient was not started on any medications for COVID-19; however, for HZ, he was prescribed famciclovir 500 mg every eight hours for seven days, and acetaminophen when needed. Since admission, his respiratory symptoms had significantly improved as well as itching and rash; nonetheless, the pain persisted and required tramadol to alleviate the discomfort. After completing seven days of famciclovir, the patient's symptoms were entirely resolved apart from mild pain at the HZ site, controlled with acetaminophen.

## Discussion

Reactivation of latent VZV in cranial-nerve or dorsal-root ganglia leads to HZ. The virus results in neural damage through migration along a particular sensory nerve. Subsequently, a vesicular rash emerges in the affected dermatome [6]. Typically, before rash development, patients may experience a preparatory itching or stinging sensation followed by vesicles pustulate. The acute phase of pain may continue up to a month, while in some cases, pain persists up to 90 days after rash resolution [6]. Aging is considered the most crucial risk factor for HZ. After acquiring a VZV infection, the T-cell immunity level starts to decline with time resulting in a reduction of protection against HZ [7]. Moreover, conditions such as patients receiving immunosuppressive medication, and those suffering from HIV infection or lymphoma also create a low T-cell level environment [8]. However, our case represented a relatively young age without risk factors insinuating a low immunity status; nonetheless, he had contracted COVID-19. In COVID-19, the minority of cases present with severe symptoms and a hyper-inflammatory state [9]. Once a body is exposed to a foreign microbe, activation of the pattern recognition receptors presented on the surface of the immune cells guides the host to commence a sepsis-like response [10]. Under these circumstances, a cytokine storm develops within several days, in addition to an extreme complement and innate immune activation. Such aggressive stimulation intensifies the inflammatory response, procuring molecular dysregulation [11]. Progression of the hyper-inflammatory state consequently causes immune cell dysfunction [12]. These outcomes create optimum habitat for HZ emergence. Unlike our case, patients with serious COVID-19, where the disease manifested drastic immunosuppression, presented with severe symptoms [13]. Nevertheless, observations on patients with mild disease have illustrated a significantly decreased T cell and CD8 levels, indicating a possibility of SARS-CoV-2 directly infecting lymphocytes, which is eventually represented in dysfunctional antiviral effects [14]. Reactivation of HZ is not frequent in COVID-19 patients, but few cases raised the concerns of the possible association. A report of two cases has demonstrated HZ reactivation preceding the emergence of respiratory symptoms in COVID-19 patients [15]. Moreover, HZ may occur in entirely asymptomatic COVID-19 patients [16]. In our case, the patient started to experience HZ two days after presenting with respiratory symptoms. We believe during the period of the COVID-19 pandemic, patients manifesting with HZ warrant healthcare workers to rule out COVID-19 and apply maximum personal protective equipment when handling such patients.

## Conclusions

We presented a case of an immunocompetent middle-aged gentleman who was admitted as a case of COVID-19 in combination with HZ. The case postulates an association between COVID-19 and reactivation of VZV in the form of HZ. In patients who present with HZ in the current pandemic of COVID-19, we believe it would be prudent to adhere to maximum precautions until the diagnosis of COVID-19 is excluded.
