# Correction: Herpes Zoster Co-Infection in an Immunocompetent Patient With COVID-19

**DOI:** 10.7759/cureus.c380

**Published:** 2025-11-18

**Authors:** Ahmed Saati, Faisal Al-Husayni, Afnan A Malibari, Anas A Bogari, Maher Alharbi

**Affiliations:** 1 Internal Medicine, National Guard Hospital, Jeddah, SAU; 2 Internal Medicine, National Guard Hospital, King Abdulaziz Medical City, Jeddah, SAU; 3 Internal Medicine, King Saud Bin Abdulaziz University for Health Sciences, Jeddah, SAU; 4 Infection Prevention and Control, King Abdulaziz Medical City, Jeddah, SAU; 5 College of Medicine, King Saud Bin Abdulaziz University for Health Sciences, Jeddah, SAU; 6 Saudi Arabia Infectious Diseases, King Abdullah International Medical Research Center, Jeddah, SAU

The affiliations for Maher Alharbi have been corrected to:

1. Infection Prevention and Control, King Abdulaziz Medical City, Jeddah, Saudi Arabia
2. College of Medicine, King Saud Bin Abdulaziz University for Health Sciences, Jeddah, Saudi Arabia
3. Saudi Arabia Infectious Diseases, King Abdullah International Medical Research Center, Jeddah, Saudi Arabia

